# Gender-specific differential expression of exosomal miRNA in synovial fluid of patients with osteoarthritis

**DOI:** 10.1038/s41598-017-01905-y

**Published:** 2017-05-17

**Authors:** Ravindra Kolhe, Monte Hunter, Siyang Liu, Ravirajsinh N. Jadeja, Chetan Pundkar, Ashis K. Mondal, Bharati Mendhe, Michelle Drewry, Mumtaz V. Rojiani, Yutao Liu, Carlos M. Isales, Robert E. Guldberg, Mark W. Hamrick, Sadanand Fulzele

**Affiliations:** 1 Departments of Pathology, Augusta University, Augusta, GA 30912 USA; 2 Departments of Orthopaedic Surgery, Augusta University, Augusta, GA 30912 USA; 3 Department of Medicine, Augusta University, Augusta, GA 30912 USA; 4 Cell Biology and Anatomy, Augusta University, Augusta, GA 30912 USA; 5 Parker H. Petit Institute for Bioengineering and Bioscience, Georgia Institute of Technology, Atlanta, Georgia, GA 30332 USA

## Abstract

The pathogenesis of osteoarthritis (OA) is poorly understood, and therapeutic approaches are limited to preventing progression of the disease. Recent studies have shown that exosomes play a vital role in cell-to-cell communication, and pathogenesis of many age-related diseases. Molecular profiling of synovial fluid derived exosomal miRNAs may increase our understanding of OA progression and may lead to the discovery of novel biomarkers and therapeutic targets. In this article we report the first characterization of exosomes miRNAs from human synovial fluid. The synovial fluid exosomes share similar characteristics (size, surface marker, miRNA content) with previously described exosomes in other body fluids. MiRNA microarray analysis showed OA specific exosomal miRNA of male and female OA. Gene Ontology (GO) analysis and Kyoto Encyclopedia of Genes and Genomes (KEGG) pathway analysis identified gender-specific target genes/signaling pathways. These pathway analyses showed that female OA specific miRNAs are estrogen responsive and target TLR (toll-like receptor) signaling pathways. Furthermore, articular chondrocytes treated with OA derived extracellular vesicles had decreased expression of anabolic genes and elevated expression of catabolic and inflammatory genes. In conclusion, synovial fluid exosomal miRNA content is altered in patients with OA and these changes are gender specific.

## Introduction

Osteoarthritis (OA) is a degenerative joint disease affecting 14% of adults aged 25 years and older and 34% of those older than 65 in the United States^1^. Importantly, the prevalence of OA is higher among women than men, and the risk for developing OA increases among women after menopause^2, 3^. The severity of OA often worsens with age, and currently no effective treatment exists to prevent the initiation and progression of the disease^4^. One of the limiting factors for ineffective OA therapeutics is partly due to an inability to diagnose early and a lack of understanding of the pathophysiology. Early diagnosis is a key factor in the prevention and management of disease. Routinely, OA is diagnosed through radiographic and physical examination, and these diagnostic methods are relatively poor for early diagnosis of OA^5^. Synovial fluid is the best source of body fluid which can be obtained by minimal invasion for OA diagnosis. Synovial fluid is a viscous body fluid found in the cavities of joints secreted by an inner layer of synovial membrane^6^. The earliest pathophysiological changes in a degenerative knee joint are first present in synovial fluid. To date, no studies have been attempted to identify differentially secreted extracellular vesicles (EVs) and their miRNA expression pattern in the synovial fluid of osteoarthritic patients. Therefore we hypothesized that, the knee joint (articular cartilage and adjacent tissue) secretes EVs containing different sets of miRNAs (in the synovial fluid) during healthy and degenerative states of disease, and that altered EVs contribute to cartilage degeneration by transporting specific miRNAs. Furthermore, identifying these special sets of miRNAs in EVs will help in early diagnosis and prevention of further damage to articular cartilage.

Almost all metabolically active cells secrete extracellular vesicles (EVs) (exosomes and micro-vesicles) including articular cartilage joint cells^7–9^. Exosomes are small (40–100 nm diameter) vesicles containing specific proteins, lipids, and RNA molecules that are secreted by cells into the extracellular space^8–12^. These EVs are now recognized to play a vital role in several age-related diseases^12–14^. Moreover, EVs are important molecules in cell-to-cell communication and take part in various metabolic activities^8–17^. A set of microRNAs (miRNAs) captured in EVs secreted by the knee joint in synovial fluid has high potential as a biomarker for early and accurate diagnosis of OA. In this study, we isolated EVs from synovial fluid of osteoarthritic (OA) and non-osteoarthritic (NON-OA) patients, captured miRNAs from these EVs and used these miRNAs to perform miRNA array to identify differentially expressed miRNAs. In doing so, we identified both differential expression of miRNAs in synovial fluid from OA patients and gender specific miRNAs within the OA population.

## Material and Methods

### Patient Samples

All methods were performed in accordance with the relevant guidelines and regulations of Augusta University and were approved by its ethical committee. De-identified discarded human synovial fluid waste samples were used for this study and does not required informed consent. All studies were performed with approval from the Augusta University Institutional Review Board (IRB). Normal and osteoarthritic human synovial fluid was obtained from the knee joints of patients undergoing arthrocentesis/total knee arthroplasty procedures. Further details are summarized in supplemental tables (Table S1). The synovial fluid was obtained from the operating room at the time of surgery, transported to the laboratory, and immediately used for exosome isolation.

### Preparation of exosome-enriched fractions

Exosome fractions were prepared by a step-wise centrifugation method^18–20^. Briefly, 1 ml synovial fluid was centrifuged at 3000 RPM for 20 minutes to remove the cell debris, and then Total Exosome Isolation Reagent (Life Technologies, Carlsbad, CA) was used to isolate exosomes as per manufacturer protocol. This protocol involved initial precipitation followed by centrifugation. After centrifugation, pellets were dissolved in 200 ul of phosphate-buffered saline (PBS) as exosome-enriched fractions.

### Nanoparticle tracking analysis

EV particle size and concentration were measured using nanoparticle tracking analysis (NTA) with ZetaView PMX 110 (Particle Metrix, Meerbusch, Germany)^18, 21, 22^ and corresponding software ZetaView. Isolated exosome samples were appropriately diluted using 1X PBS buffer (Life Technologies, Carlsbad, CA, USA) to measure the particle size and concentration. NTA measurement was recorded and analyzed at 11 different positions. The ZetaView system was calibrated using 100 nm polystyrene particles and temperature was maintained at 23 °C.

### Electron microscopy (EM) and Immuno-gold labeling of EVs

Transmission electron microscopy and an immunogold labeling method were used to validate our isolation approach. EM imaging of EVs preparations was performed as described previously^18, 23, 24^, with some modifications. Briefly, EVs were fixed in 1% glutaraldehyde for 2–4 hours. The fixed EVs then layered and dried on Formvar coated 200 mesh copper grids (Polysciences, Inc. PA, USA). Grids were then stained with 1% uranyl acetate in water. Grids were allowed to air dry before being examined in a JEM 1230 transmission electron microscope (JEOL USA Inc., Peabody MA) at 110 kV and imaged with an UltraScan 4000 CCD camera & First Light Digital Camera Controller (Gatan Inc., Pleasanton, CA.) For Immuno-gold labeling, established surface marker for extracellular vesicles CD9 was used. The samples were probed using antibodies diluted 1:100 to CD9 (Santa Cruz, rabbit polyclonal). Exosome samples were fixed in 4% paraformaldehyde, 2% glutaraldehyde in 0.1 M cacodylate buffer pH 7.4 overnight. The microvesicles (20 µl) was applied to a carbon-Formvar coated 200 mesh copper grid and allowed to stand 30–60 seconds, and the excess wicked off onto Whatman filter paper. Grids were floated on drops of 1.4 nm anti-Rabbit nanogold (Nanoprobes, Inc.) diluted 1:1000 in blocking buffer for 1 hour. Grids were enhanced 1 minute in HQ Silver (gold enhancement reagent, Nanoprobes, Inc.) and rinsed in ice cold DI H_2_O to stop enhancement and then negatively stained in 2% aqueous Uranyl Acetate and wicked dry. Images were captures as described above.

### Western blot analysis

Extracellular vesicles were lysed in RIPA buffer containing proteinase inhibitor cocktail (Sigma) followed by quantification of total protein concentration using a Bradford assay (Bio-Rad Laboratories) according to the manufacturer’s protocol. Equivalent amounts of total lysate were subjected to SDS-PAGE and transferred to nitrocellulose membranes. Membranes were incubated with an antibody against CD81, CD63 and TSG101 (Santa Cruz, CA) overnight at 4 °C, followed by incubation with HRP-conjugated goat anti-rabbit IgG antibody. Proteins were visualized with an ECL Western blot detection system (Thermo Scientific, Waltham, MA).

### EV miRNA isolation and microarray profiling

miRNAs were isolated from extracellular vesicles using Qiagen miRNeasy Kit according to manufacturer’s protocol. The concentration of miRNA was determined using a NanoDrop spectrophotometer (Thermo Scientific) and the quality of miRNA was analyzed using an Agilent 2100 Bioanalyzer. Microarrays were performed on miRNA using an Affymetrix GeneChip® miRNA 4.0 Array at the Integrated Genomics Core, Augusta University, GA. Details of the procedure can be found online at http://www.augusta.edu/cancer/research/shared/genomics/index.php. The miRNA profile was analyzed for supervised as well as unsupervised hierarchical clustering to generate heat maps between male and female OA and NON-OA samples.

### Normalization, statistical analysis and pathway analysis of microRNA array

miRNA expressions were compared between the groups. The housekeeping genes (snoRNA251, snoRNA202, snoRNA142, and U6) in the miRNA PCR arrays, were averaged as the endogenous control and the NON-OA group was used as external control for normalization of samples. T-tests were used to calculate the p value to determine the significant difference for miRNA expression between the groups (OA and NON-OA). The p value cutoff of 0.05 and the miRNAs with a fold change above 1.5 were considered differentially expressed for further analyses. Gene Ontology (GO) and KEGG signaling pathway analyses were performed using DIANA-miRPath v 3.0 (http://diana.imis.athena-innovation.gr/DianaTools/index.php) on differentially expressed microRNAs target genes^25^. GO and KEGG word clouds were generated using the online Wordle software (www.wordle.net). Principal component analysis (PCA) was performed between male and female OA samples.

### Validation of miRNAs using real-time PCR

Real-time PCR was performed on randomly selected miRNAs (miR-6878-3p, miR-210-5p, miR-16-2-3p,miR-26a-5p, miR-146a-5p and miR-6821-5p) to validate miRNA array data in age matched male (NON-OA, n = 9 and OA, n = 15) and female donor samples (NON-OA, n = 9 and OA, n = 16-18). EV miRNAs were isolated as described above. Two hundred nanograms of enriched miRNAs were converted into cDNA using miScript II RT Kit (from Qiagen). Fifty pictograms of cDNA were amplified in each qRT-PCR using SYBR Green I and miR specific primers. The real-time qRT-PCR was performed on a Bio-Rad MyiQ machine with following cycling parameters: 95 °C for 10 mins, then 40 cycles of 95 °C for 15 s, 60 °C for 30 s and 72 °C for 30 s. SYBR Green fluorescence was recorded from every well during the annealing step of each cycle. A melt curve was generated to analyze the purity of amplification products. The average of RNU6 (RNA, U6 small nuclear 2) and SNORD (small nucleolar RNA, C/D box) was used as normalization reference genes for miRs. Relative expression of miRNA was evaluated by using the comparative CT method (ΔΔCt).

### OA-derived EVs regulate articular chondrocyte gene expression and cell survival

Female articular chondrocytes (AC) were isolated from healthy donors as per published method^26, 27^. The AC cells were cultured in 24 well plates and treated with OA and NON-OA synovial fluid derived EVs (40 µg/ml) separately with 1% FBS (exosome free) media for 48 hrs. We pulled down exosomes from 6 female donor’s [age matched in each group (6-OA and 6-NON-OA separately] to perform this experiment. Collagen type II, aggrecan, IL-6, and TNFα gene expressions were performed using real time PCR [Primers details in supplemental tables S2 (Table S2)]. MTT assays were performed on cells to analyze cell survivability and gelatin zymography was performed on cell culture supernatant for MMPs activity. MTT assay^28^ and gelatin zymography^29^ were performed as per published methods.

### *In vitro* miRNA transfection and estrogen inhibitory studies

For miRNA transfection studies, AC cells were cultured in 24 well plates. Scrambled (Negative control) miRNA and miRNA mimics for miR-181d-3p, miR-185-5p and miR-7107-5p were purchased from QIAGEN. Lipofectamine 2000 was utilized for transfecting miRNA mimics (20 nM) in AC cells according to the manufacturer’s instructions and our published protocol^28^. After 24 hrs, transfected cells were used for mRNA isolation and followed by real time PCR.

To identify the role of estrogen signaling on EVs miRNA cargo, primary synovial fibroblast cells were isolated as previously described by Zimmermann *et al*.^30^. Synovial fibroblast were plated on 100 mm plate cultured in phenol red–free culture medium containing 5% charcoal-treated fetal bovine serum for 24 hrs to eliminate the estrogen-like effect of phenol red and fetal bovine serum. The cells were then cultured with or without letrozole (20 nM) for 24 hrs in 1% charcoal-treated fetal bovine serum. Letrozole is a non-steroid aromatase inhibitor, which selectively prevent estrogen synthesis^31^. Exosomes were isolated after 24 hrs of treatment as per described above, followed by miRNA isolation and real-time PCR on selected miRNAs.

### Synovial fluid derived extracellular vesicles RNA are endocytosed by chondrocytes

Extracellular vesicles isolated from OA patient’s synovial fluid were labeled with Exo-Red dye (Cat: EXOR100A-1, SBI, CA, USA). Exo-Red stain fluorescently-labels single-stranded RNA cargo of exosomes. Labeling of exosomal RNA was performed as per manufactures protocol. Briefly, 10 μL of Exo-Red was added to 100 μL of extracellular vesicles suspension and incubated for 15 min at 37 °C. The labeled exosomes were precipitated using ExoQuick-tissue culture reagent. Articular chondrocyte (AC) cells were treated with labeled exosomes for 12 hrs and endocytosis was confirmed by fluorescence microscope.

### Statistical analysis

Data are presented as fold-changes or percentages with mean ± SEM as indicated in the figure legends. GraphPad Prism 5 (La Jolla, CA) was utilized to perform unpaired t-tests as appropriate. A p-value of 0.05 was considered significant.

## Results

### Human synovial fluid contains high concentration of extracellular vesicles

**Figure 1:**
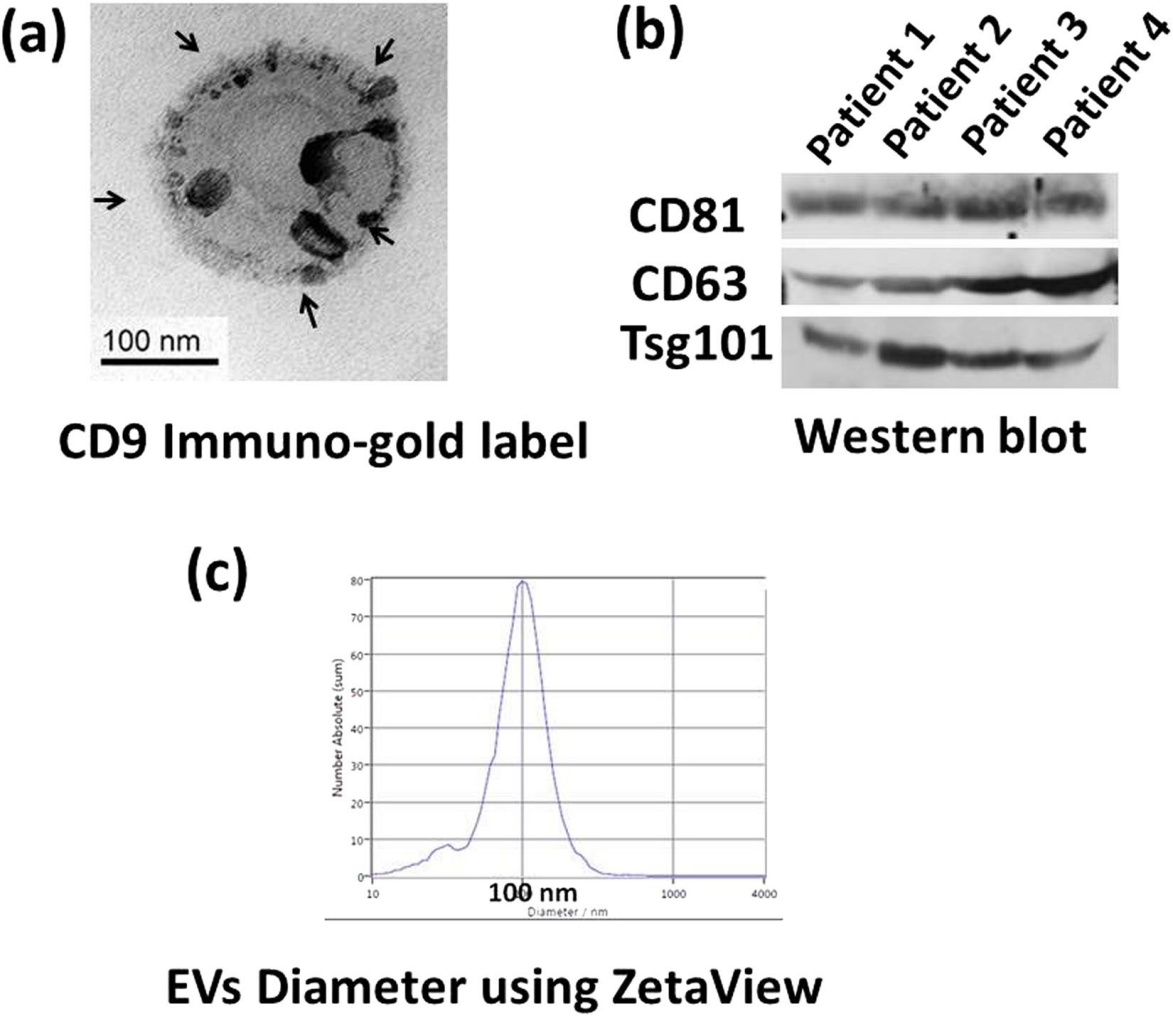
Characterization of synovial fluid derived-EVs. (**a**) Transmission electron microscope images of immuno-gold labeled of EVs showing positive labeling for the exosome markers CD9 (arrows). (**b**) Western blot demonstrating the expression of CD81, CD63, and TSG101 in four different patients and (**c**) Particle size distribution EVs (average size 100 nm), measured by ZetaView® Particle Tracking Analyzer. Cropped gels were displayed and full-length gels and blot were included in the Supplementary Information file.

We isolated EVs from synovial fluid of non-osteoarthritic (NON-OA) and OA patients utilizing Total Exosome Isolation Reagent, precipitation and centrifugation. This method is recognized to produce the high purity, quality, and yield of extracellular vesicles isolated from biological fluids^18–20^. To confirm the presence of EVs, they were examined using electron microscopy, immuno-gold staining and western blot. Electron micrographs revealed that the isolated EV particles consisted of primarily round shaped vesicles (Fig. 1a). Immuno-gold staining showed positive staining for exosome markers CD9 (Fig. 1a). Western blot analysis showed band of exosome markers Tsg101, CD63 and CD81 (Fig. 1b). Nanoparticle tracking analysis was performed using the ZetaView instrument from MicroTrac. These data showed that vesicles isolated from synovial fluid are in the ~100 (±10) nm diameter size ranges (Fig. 1c), consistent with the known size of EVs^7–12^.

### OA alters synovial EV miRNA expression in a gender-specific manner

**Figure 2:**
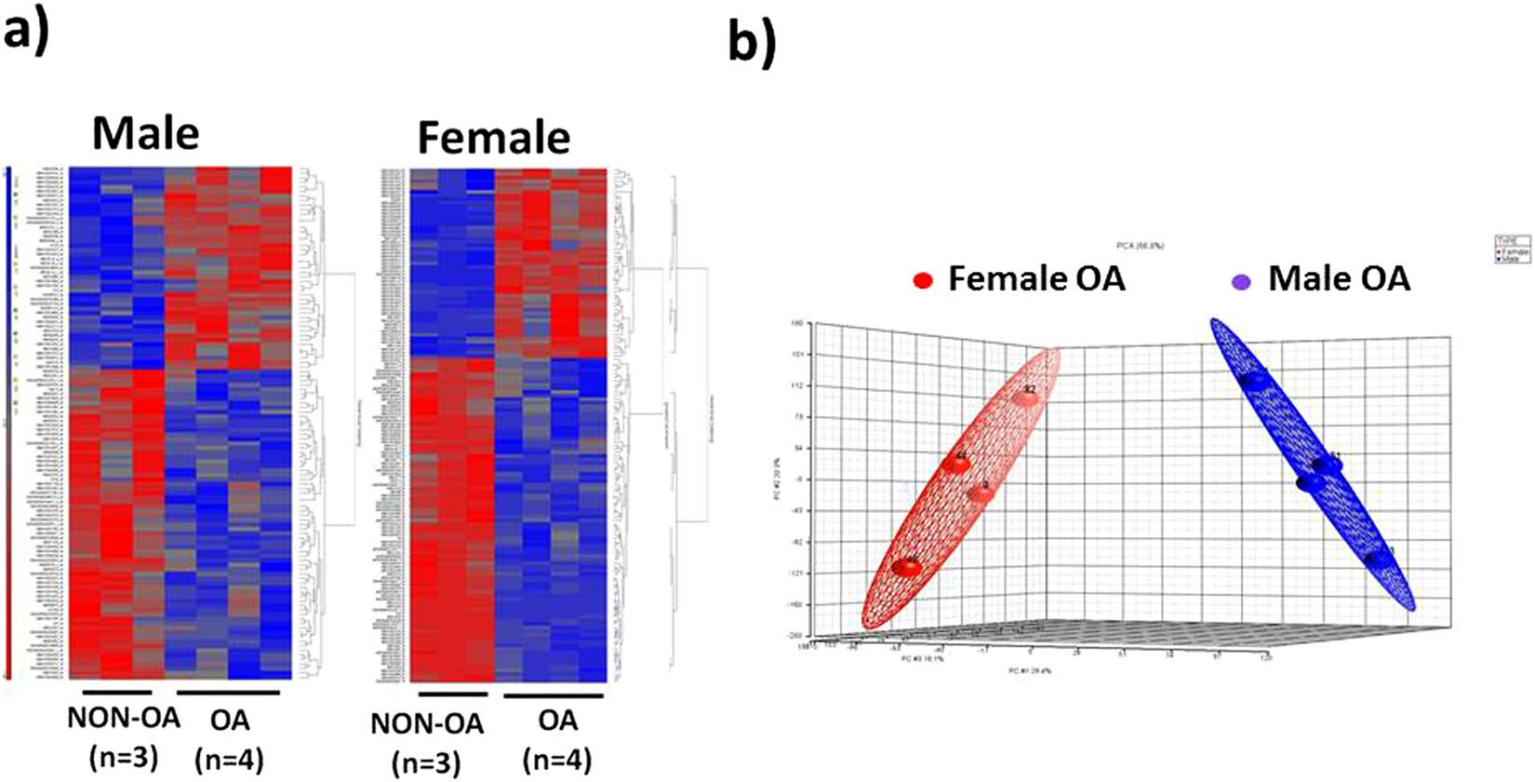
Alteration in the miRNAs carried by EVs in osteoarthritis synovial fluid. Heat-map of (**a**) male OA (n = 4) and NON-OA (n = 3) and female OA (n = 4) and Non-OA (n = 3). (**b**) Principle component analysis (PCA) mapping of female OA and male OA profiling. Female OA group (indicated by red color) was clustered distinctly from male group (indicated by blue color).

The size and concentration of EVs are almost similar between OA and NON-OA patient’s synovial fluid. Synovial fluid derived EVs were used to isolate miRNAs using Qiagen miRNeasy kit. Bioanalyzer traces show these vesicles are highly enriched in miRNAs. Microarrays were performed using an Affymetrix GeneChip® miRNA 4.0 Array at the Integrated Genomics Core, Augusta University, GA to identify OA specific miRNAs. Age matched NON-OA (n = 3) and OA (n = 4) of each gender with no significant co-morbidities (diabetes, cardiovascular, HIV, or other diseases) were used to perform miRNA array. We found that miRNA content of the EVs differ between OA and NON-OA groups (Tables 1 and 2). In male 69 miRNAs were significantly down-regulated and 45 miRNAs were up-regulated where as in female, 91 miRNAs down-regulated and 53 miRNAs up-regulated. Interestingly, the data showed gender specific differences in miRNA content in osteoarthritis EVs. The principal component analysis (PCA) plots also suggest a clear separation of differentially expressed miRNAs in male and female OA (Fig. 2b). The supervised (Fig. 2a) heat map clustering showed clear difference between OA and NON-OA in both male female samples. Un-supervised (Supplementary Fig. S1) heat map clustering showed trends of difference between OA and NON-OA of both genders. MiR-504-3p is the only common miRNA up-regulated in both male and female OA patients. Tables 1 and 2 shows selected miRNAs dysregulated in EVs isolated from OA synovial fluid.

**Table 1:** Selected miRNAs differentially regulated in male.

Male (NON-OA vs OA)		
miRNA	Fold Change	p-value
hsa-miR-6878-3p	−1.69735	2.37E-05
hsa-miR-4797-5p	−1.57955	0.0351181
hsa-miR-6828-5p	−1.53631	0.0288964
hsa-miR-6076	−1.53465	0.00670878
hsa-mir-561	−1.53093	0.000766648
hsa-miR-6753-3p	−1.5267	0.0424525
hsa-miR-934	−1.51141	0.0049018
hsa-miR-5002-5p	−1.47844	0.0218813
hsa-miR-519b-3p	−1.46841	0.0206755
hsa-miR-661	−1.4576	0.032959
hsa-miR-211-5p	−1.45526	0.037896
hsa-mir-2114	−1.44728	0.00512611
hsa-miR-5197-5p	−1.41533	0.0310416
hsa-miR-2355-5p	−1.40993	0.0194376
hsa-miR-221-5p	−1.4087	0.00996333
hsa-miR-522-3p	−1.40596	0.046033
hsa-mir-199b	−1.39955	0.0469109
hsa-mir-181a-1	1.36981	0.00181767
hsa-miR-191-5p	1.39754	0.0329004
hsa-miR-342-5p	1.39801	0.00995949
hsa-miR-6736-5p	1.39823	0.0109007
hsa-miR-6763-3p	1.42149	0.0240226
hsa-mir-26b	1.42652	7.02E-05
hsa-miR-34a-3p	1.43162	0.0228724
hsa-miR-2276-5p	1.43541	0.0488764
hsa-miR-4714-5p	1.44909	0.0373723
hsa-miR-210-5p	1.46205	0.0043617
hsa-miR-504-3p	1.48862	0.0171264
hsa-mir-6743	1.57725	0.00391291
hsa-miR-5090	1.62259	0.0311708
hsa-miR-4749-5p	1.67441	0.0469231

**Table 2:** Selected miRNAs differentially regulated in female OA.

Female (NON-OA vs OA)		
miRNA	Fold Change	p-value
hsa-miR-24-3p	−6.07434	0.00270441
hsa-miR-23a-3p	−5.03367	0.0239493
hsa-miR-26a-5p	−4.04088	0.000363269
hsa-miR-4487	−3.3772	0.0137512
hsa-miR-6821-5p	−3.29715	3.81E-05
hsa-miR-4508	−3.05877	0.00283766
hsa-miR-6790-5p	−2.9154	2.46E-05
hsa-miR-4654	−2.77	0.000158951
hsa-miR-6858-5p	−2.66954	0.0159022
hsa-miR-5100	−2.49473	0.00257262
hsa-miR-6732-5p	−2.4613	0.00125506
hsa-miR-4740-5p	−2.41614	5.74E-05
hsa-miR-4707-5p	−2.33459	0.018536
hsa-miR-328-5p	−2.30093	0.00187988
hsa-miR-6824-5p	−2.22158	0.00120888
hsa-miR-7158-5p	−2.14365	6.44E-05
hsa-mir-4317	−2.09132	8.28E-07
hsa-miR-146a-5p	−1.71279	0.000647412
hsa-miR-504-3p	1.72003	0.0001195
hsa-miR-185-5p	1.98088	0.0414396
hsa-miR-181d-3p	1.9922	0.00103063
hsa-mir-4716	1.99678	0.0041666
hsa-miR-3940-3p	2.01176	0.00043868
hsa-miR-890	2.08514	0.000211651
hsa-miR-4454	2.15815	0.0374761
hsa-miR-4459	2.16699	0.0114644
hsa-mir-4640	2.19009	0.000688567
hsa-miR-4305	2.19473	0.0104292
hsa-miR-16-2-3p	2.34654	2.38E-05
hsa-miR-155-3p	2.47419	0.0189391
hsa-miR-4274	2.56957	0.030786
hsa-miR-6865-3p	2.97735	0.00818417
hsa-miR-4532	3.22896	0.00244072
hsa-miR-7107-5p	3.30428	0.0472756

**Figure 3:**
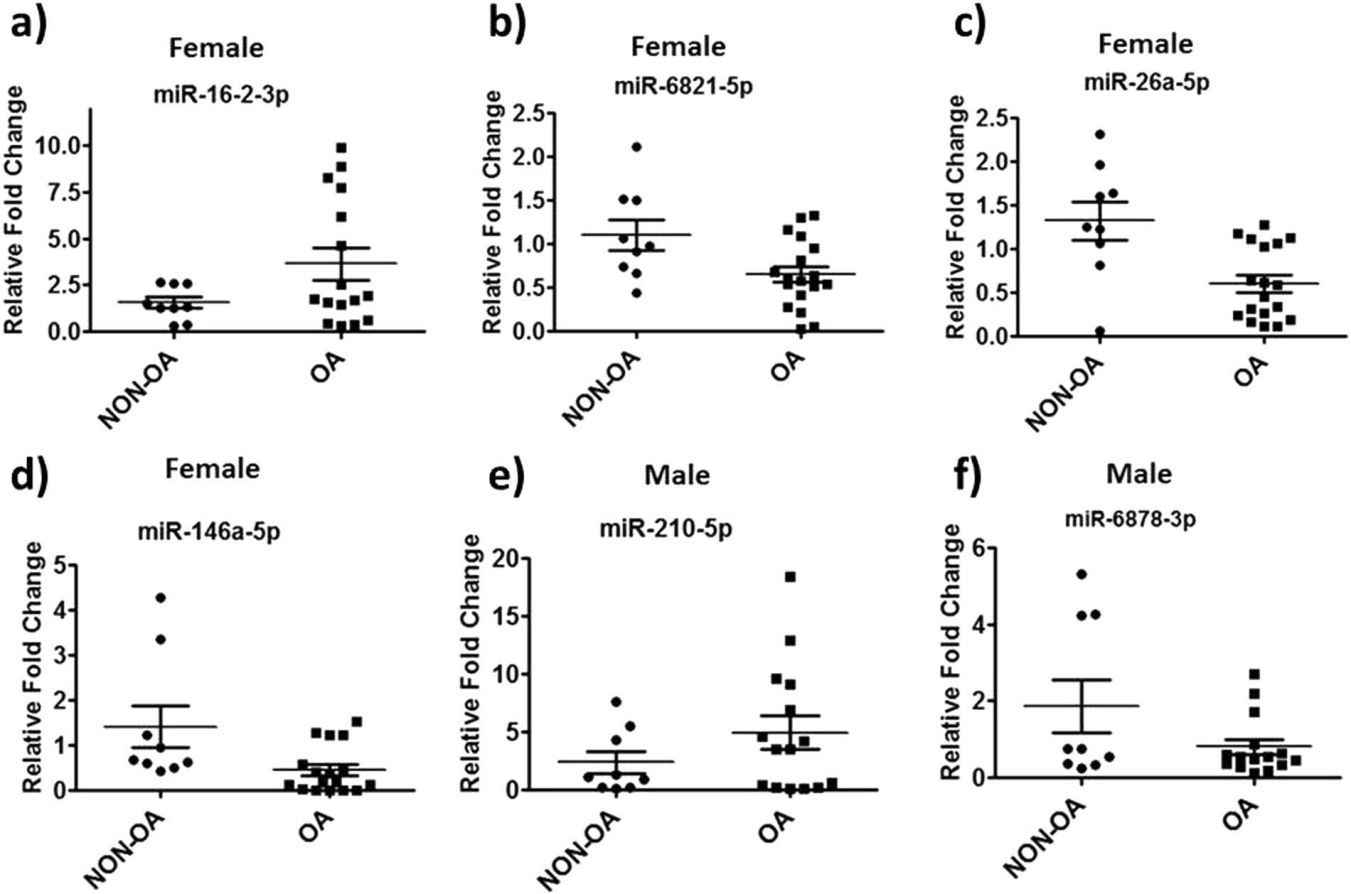
OA alters the miRNAs content in synovial derived EVs in gender specific manner. Real-time PCR validation showing change in miRNA expression in female samples [n = 9 (NON-OA) and n = 16–18 (OA)] (**a**) miR-16-2-3p (p = 0.085), **(b**) miR-6821-5p (p = 0.017), (**c**) miR-26a-5p (p = 0.01), and d) miR-146a-5p (p = 0.01830) and e) miR-210-5p (p = 0.2033) and f) miR-6878-3p (p = 0.0823) in male samples [n = 9 (NON-OA) and n = 15 (OA)].

To further verify the result obtained from miRNA microarrays, we performed real-time PCR on randomly selected miRNAs to validate miRNA alterations in age matched male (NON-OA, n = 9 and OA, n = 15) and female donor samples (NON-OA, n = 9 and OA, n = 16-18). miRNA real time PCR showed similar changes as noted in miRNA array (Fig. 3). In female samples, we found that miR-16-2-3p (*p* = *0*.*085*) up-regulated and miR-26a-5p (*p* = *0*.*01*), miR-146a-5p (*p* = *0*.*01830*) and miR-6821-5p (*p* = *0*.*017*) down-regulated. In male samples, we found that miR-6878-3p (*p* = *0*.*0823*) down-regulated and miR-210-5p (*p* = *0*.*2033*) up-regulated similar to our array findings. Our data therefore suggest that miRNAs carried by extracellular vesicles in the synovial fluid are significantly altered with the osteoarthritic condition and are highly gender specific.

### GO and KEGG pathway analysis of differentially expressed miRNAs

To identify functions of the differentially expressed miRNAs, Kyoto Encyclopedia of Genes and Genomes (KEGG) pathway annotation and GO analysis were performed.

The KEGG annotation analysis for down-regulated miRNAs in OA for both male and female showed glycan degradation, cell adhesion molecules (CAMs) and mucin type O-Glycan biosynthesis were involved whereas for up-regulated miRNAs, thyroid hormone synthesis, biotin metabolism and amphetamine addiction signaling were involved (Table S3). The KEGG annotation analysis for female OA patients showed involvement in ovarian steroidogenesis signaling and estrogen signaling pathway. Details of KEGG annotation analysis for both male and female are shown in Supplementary Table S3.

**Figure 4:**
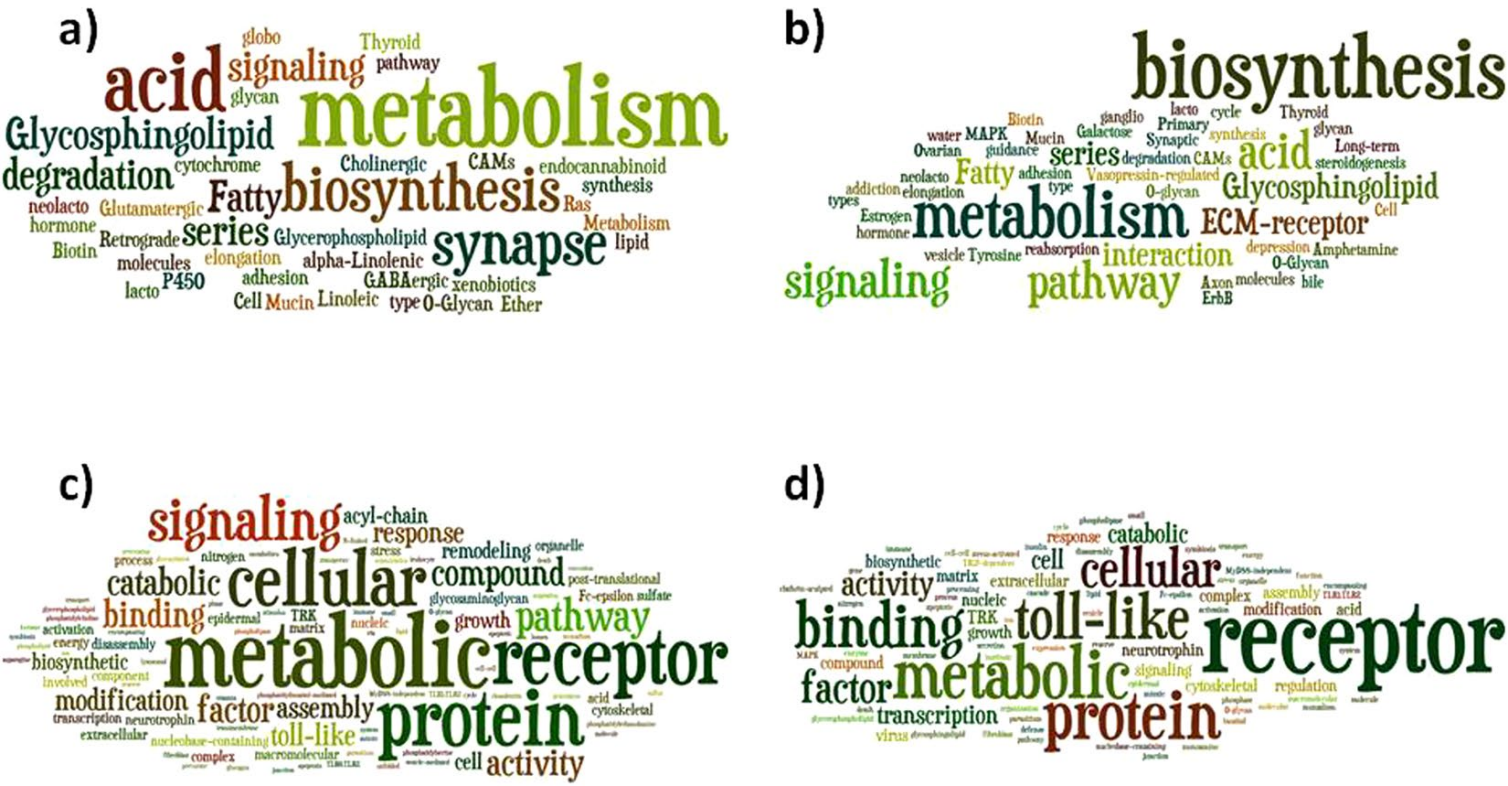
Wordle-based clouds for combine differentially (up and down-regulated) regulated miRNAs of (**a**) KEGG male, (**b**) KEGG female, (**c**) GO male and (**d**) GO female analysis. Word clouds demonstrating the font size depending on relative word frequencies in KEGG and GO analysis.

GO analysis showed more than 45 biological processes were associated with the down-regulated and up-regulated miRNAs both in male and female OA patients (Table S4). Male and female OA showed some common biological processes such as organelle, ion binding, biosynthetic process, cellular protein metabolic process and others (Table S4). Additionally, we found differentially regulated GO pathways in male and females. The male OA up-regulated miRNAs showed more than 53 GO biological processes altered whereas female showed only about 22. The most differentially regulated GO biological processes by up-regulated miRNAs in males are cell-cell signaling, immune system process, response to stress, and number of TLR signaling pathways whereas in females; they are cellular lipid metabolic process, mitotic cell cycle and clathrin-sculpted monoamine transport vesicle membrane signaling. Similarly, the down-regulated miRNAs showed a number of GO biological processes altered in OA. The female OA down-regulated miRNAs showed more than 70 GO biological processes altered whereas male showed only about 49. The most differentially regulated GO biological processes by down-regulated miRNAs in females are cell-cell signaling, immune system process, innate immune response and number of TLR signaling pathways whereas in males; they are various metabolic process (such as glycosaminoglycan, chondroitin sulfate, phospholipid, keratan sulfate), extracellular matrix organization, cellular component assembly and others. Details of GO analysis for both male and female are shown in (Table S4). Wordle-based clouds were generated for both KEGG and GO analysis (Fig. 4). Word clouds demonstrate the font size depending on relative word frequencies in KEGG and GO analysis.

### MicroRNAs regulate expression of estrogen signaling genes in articular chondrocytes

**Figure 5:**
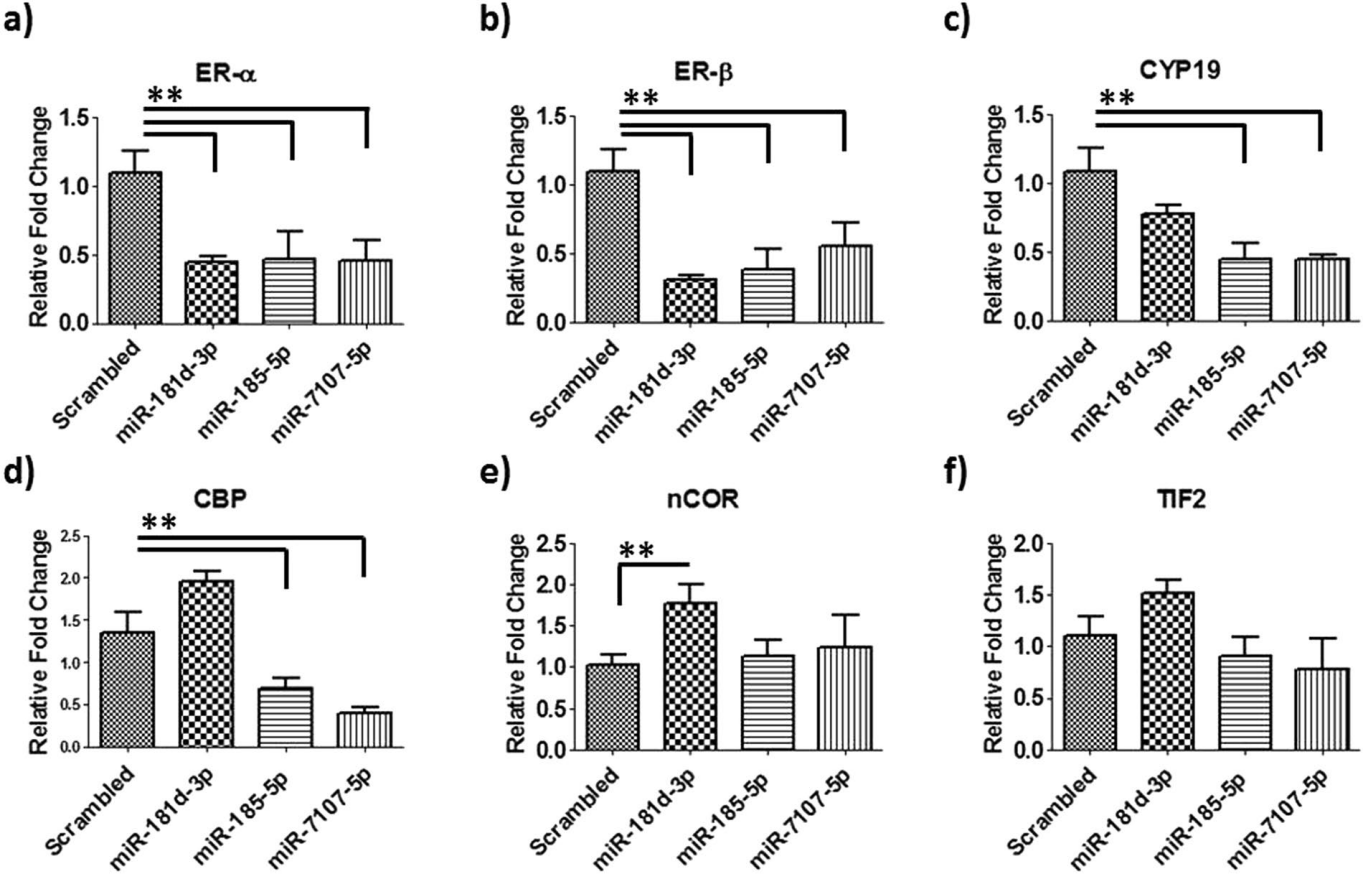
MicroRNAs regulate expression of estrogen signaling genes in articular chondrocytes. MiRNAs (181d-3p, miR-185-5p, miR-7107-5p) regulates (**a**) ER-α (estrogen receptor- α), (**b**) ER-β estrogen receptor- β), (**c**) CYP19 (aromatase cytochrome P450), (**d**) CREB-binding protein, (**e**) nCOR (Nuclear Receptor Co-repressor) and (**f**) TIF2 (Transcriptional Intermediary Factor 2) in human articular chondrocytes. Data (means ± SD, n = 6) are represented as the fold change in expression compared with control (*p < 0.05, **p < 0.01).

To determine whether OA related miRNAs are involved in estrogen-mediated gene regulation of articular chondrocytes, the cells were treated with miRNA mimics (181d-3p, miR-185-5p, miR-7107-5p) for 24 h and estrogen signaling pathway genes were analyzed. Quantitative real time PCR showed that miRNA mimics of 181d-3p and miR-185-5p significantly (p < 0.01) down-regulated the expression of estrogen receptor-α, estrogen receptor-β, and aromatase cytochrome P450. MicroRNA-7107-5p mimic significantly (p < 0.01) down-regulated expression of estrogen receptor-α and estrogen receptor-β, but produced no change in aromatase cytochrome P450 (CYP19A1) (Fig. 5). We also found that miR-185-5p and miR-7107-5p significantly (p < 0.05) down regulated expression of CREB-binding protein whereas miR-181d-3p did not show significant change in expression of CREB-binding protein (Fig. 5). Surprisingly, miR-181d-3p significantly (p < 0.01) up-regulated nuclear receptor co-repressor (n-COR) and no change in TIF2 (Transcriptional Intermediary Factor 2).The miR-185-5p and miR-7107-5p mimics did not show any changes in n-COR and TIF2 gene expressions (Fig. 5).

### Estrogen signaling regulates EVs miRNA cargo of primary synovial fibroblast cells

**Figure 6:**
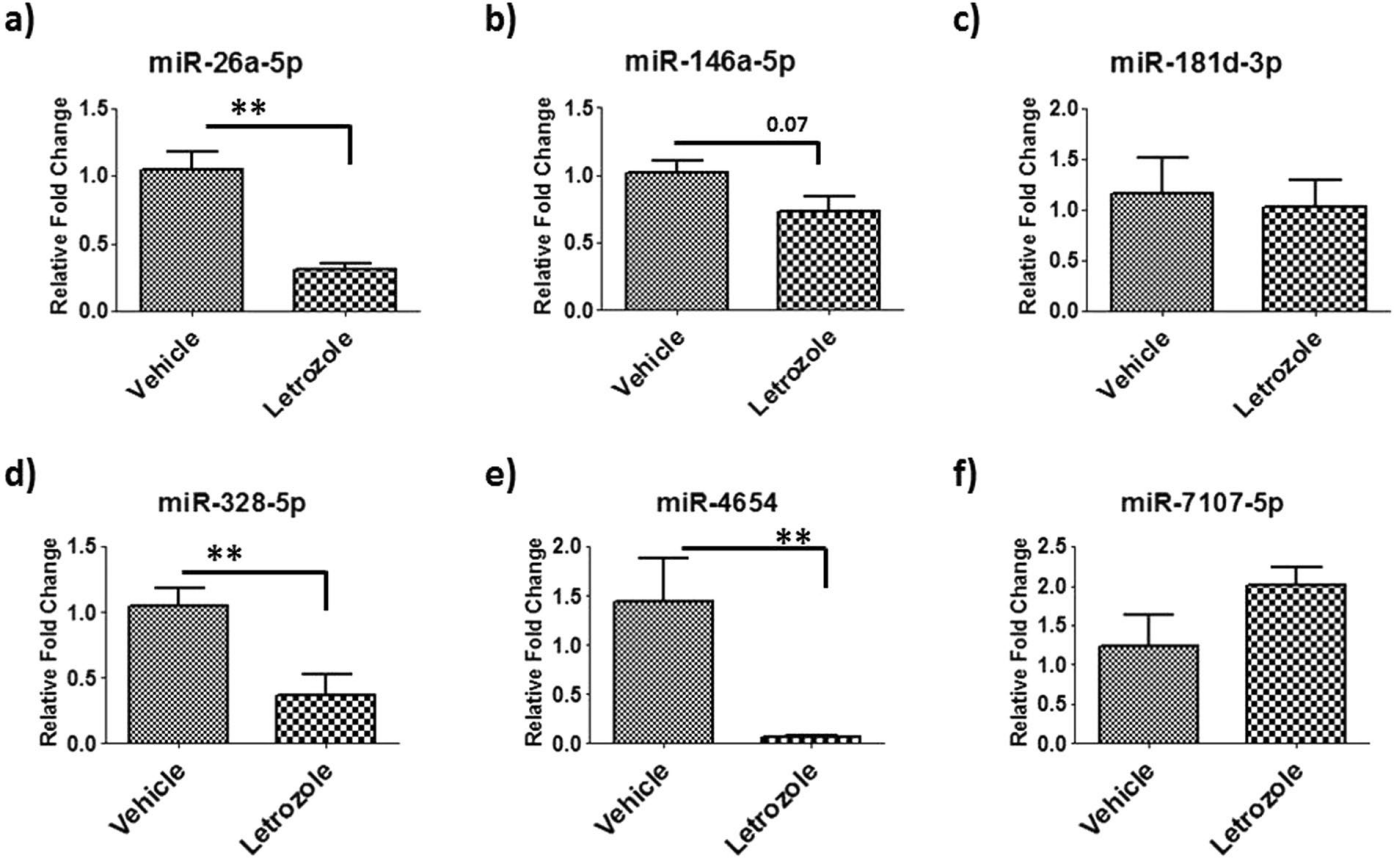
Estrogen signaling regulates EVs miRNA cargo of primary synovial fibroblast cells. Estrogen inhibitor (Letrozole) down-regulates (**a**) miR-26a-5p, (**b**) miR-146a-5p, (**c**) miR-328-5p, (**d**) miR-4654 and up-regulated (**e**) miR-7107-5p in EVs cargo of human synovial fibroblast cells. Data (means ± SD, n = 4–6,) are represented as the fold change in expression compared with control (*p < 0.05, **p < 0.01).

We hypothesized that estrogen might play an important role in EV-derived miRNA in the knee joint. To test this hypothesis, we treated synovial-fibroblast like cells with aromatase inhibitor and analyzed miRNA content of EVs secreted from cells. Synovial membrane-derived cells (synovial fibroblasts) were used because of their established role in osteoarthritis progression^32^. Our approach was based on our understanding that exosomes secreted from one cell type (synovial fibroblast) release their cargo into target cells (articular cartilage) and trigger downstream effect/signaling pathways. Our results show that treatment with an aromatase inhibitor significantly decreases content of miR-26a-5p (p < 0.01), miR-146a-5p (p < 0.07), miR-328-5p (p < 0.01), and miR-4654 (p < 0.01) (Fig. 6). We did not find any significant changes in content of miR-181d-3p and miR-7107-5p in EVs cargo.

### RNA from synovial fluid-derived extracellular vesicles is endocytosed by chondrocytes

**Figure 7:**
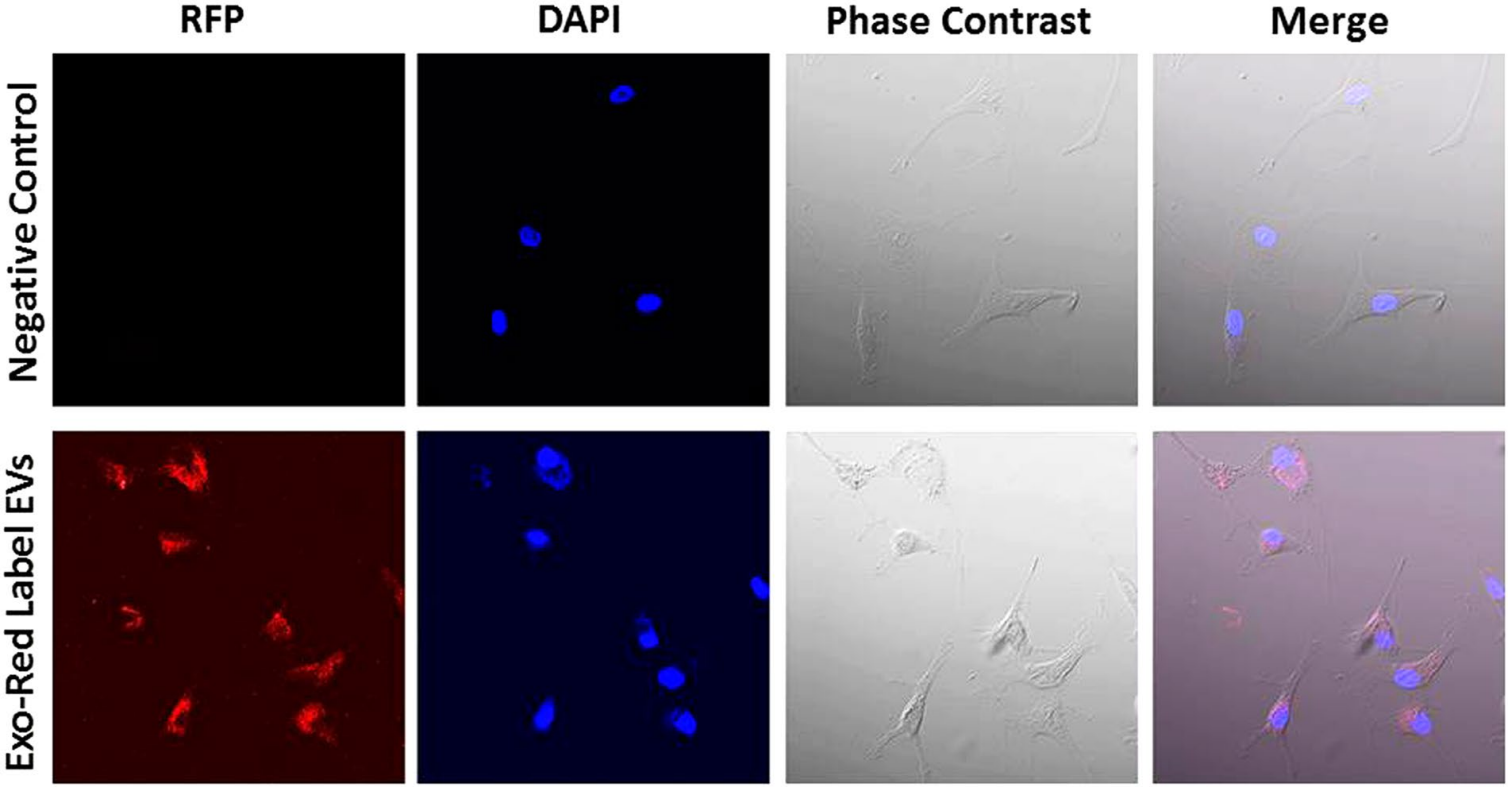
Human articular chondrocytes cells accumulate synovial fluid derived EVs. Human articular chondrocytes cells were treated with mock (negative control) and synovial fluid derived EVs labeled with Exo-Red dye. Chondrocytes cells endocytose Exo-Red-labeled exosomes.

We tested the hypothesis that extracellular vesicles isolated from OA patients’ synovial fluid might be taken up by human chondrocytes. To confirm the endocytosis process, human AC cells were treated with Exo-Red labelled (Cat: EXOR100A-1, SBI, USA) EVs. We found that human chondrocytes readily endocytose these EVs and their RNAs as indicated by positive red-fluorescence staining (Fig. 7).

### OA-derived EVs regulate articular chondrocyte gene expression and cell survival

**Figure 8:**
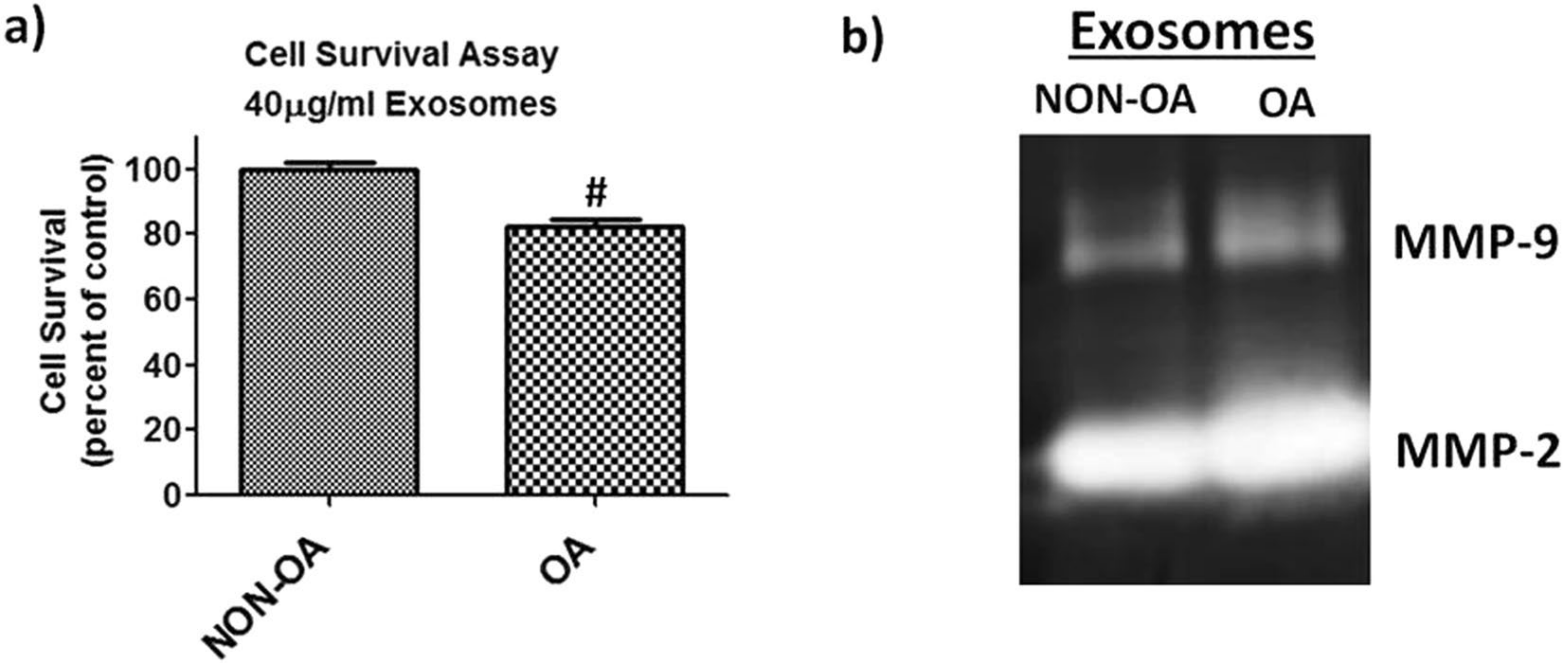
OA derived EVs decrease cell survival and increase MMPs activity. (**a**) Healthy articular chondrocytes treated with NON-OA and OA derived EVs exosomes at concentrations of 40 µg/ml for 48 hrs followed by MTT assay (n = 8/group ^#^P < .001). (**b**) Representative SDS-PAGE zymography reveals OA derived EVs enhanced MMPs activity in the cell culture supernatant. Gelatin zymography shows MMP-9 and MMP-2 activities in the culture medium of NON-OA and OA treated exosomes (40 µg/ml) for 48 hrs.

**Figure 9:**
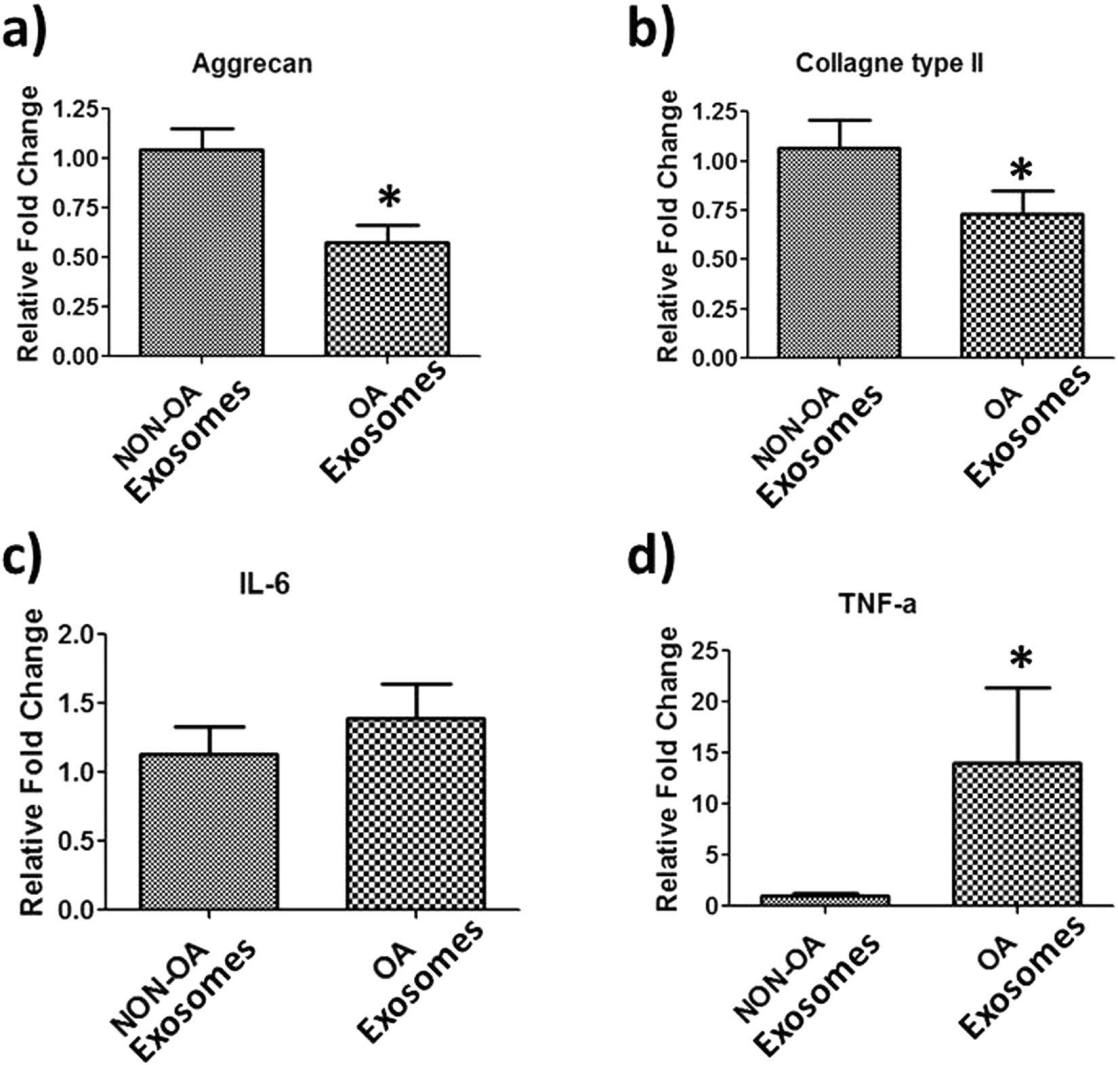
OA derived EVs regulate articular chondrocytes gene expression. Healthy articular chondrocytes treated with NON-OA and OA derived EVs exosomes at concentrations of 40 µg/ml for 48 hrs followed by RT-PCR, (**a**) Aggrecan (**b**) Collagen type II (**c**) IL-6 and (**d**) TNF-α. Data (means ± SD, n = 6) are represented as the fold change in expression compared with control (*p < 0.05).

To gain further insight into the pathogenesis of OA, we examined the effect of EVs on articular chondrocytes. We treated articular chondrocytes from healthy females with EVs derived from patients with or without OA. Cell survival is a crucial element in OA pathogenesis. We therefore performed cell survival assays and gene expression analysis after treatment with EVs. Our results showed a significant decrease in cell survival with treatment using OA-derived EVs (*P < 0.05) (Fig. 8a). Moreover, our zymography results showed significant increase of MMPs (MMP-2 and MMP-9) activity in cell culture supernatant (Fig. 8b). The gene expression of extracellular matrix synthesis genes (Aggrecan, COL-II) decreased whereas inflammatory (IL-6, TNFα) gene expression significantly increased (Fig. 9). Overall, the treatment of OA-derived EVs decrease chondrocyte survival and anabolic gene expression and increase catabolic gene expression. These data indicate that OA-derived EVs play a vital role in chondrocyte pathophysiology.

## Discussion

Synovial fluid is useful for monitoring pathophysiological changes in the joint space because of its direct and intimate relationship with synovial membrane, articular cartilage and other tissue types of knee joint. Recently, a number of studies investigated synovial fluid content, such as protein, cellular metabolites and miRNAs for its role in pathophysiology and possible biomarkers^33, 34^. Skriner *et al*. previously reported that synovial exosomes of rheumatoid arthritis (RA) patients with reactive arthritis, and patients with osteoarthritis, contain citrullinated proteins^7^. Our study focused on synovial fluid extracellular vesicle content, specifically on capture-miRNAs carried within these EVs. Exosomes are 40–100 nm diameter packaged vesicles containing specific proteins, lipids, and/or genetic material that are secreted by almost all metabolically active cells^7–12^. Exosomes are cellular byproducts which reflect the pathophysiological changes that occur nearby or in its surrounding environment, which makes them particularly useful for the development of highly sensitive diagnostic tools for monitoring disease progression^7–19^. To the best of our knowledge, our study is the first to characterize EVs miRNAs cargo from synovial fluid of non-osteoarthritic (NON-OA) and OA patients. Nanoparticle tracking analysis shows that vesicles isolated from synovial fluid are in the ~100 (±10) nm diameter size ranges, consistent with the known size of EVs^9, 10, 14–18^. The size and concentration of EVs are similar between OA and NON-OA patients’ synovial fluid and these extra cellular vesicles are highly enriched in miRNAs.

EVs are released from the cells in response to surrounding environment, external stimuli and physiological status of cells^9, 35^. We hypothesized that synovial fluid-derived exosomal miRNA content varies with degenerative status of knee joints. miRNA arrays were performed on OA and non-OA synovial fluid derived exosomal miRNA. We have profiled the number of unique EV miRNAs in synovial fluid from the arthritis compared to those with control. Some of the miRNAs showing differential expression in OA, (such as miR-146a, miR-26a, and miR-210) had previously been associated with cartilage pathophysiology and other musculoskeletal diseases^36–40^. Our studies revealed a unique OA specific miRNA (miRNA-504) in both genders.

The prevalence of OA is higher among women than men, and the risk of developing OA increases among women after menopause^3, 41–45^. Considering gender-dependent differences in the prevalence of OA, we analyzed array data separately to identify novel gender-specific exosomal miRNA. Interestingly, we identified novel gender specific miRNAs in the OA population. We noted that the female OA group has a higher number of miRNAs that are differentially (up and down) regulated than males. The miRNA array data was further validated using additional samples of selected miRNAs. We demonstrated similar results, indicating that these exosomal miRNAs can be used as gender specific biomarkers for diagnosis of OA. Exosomal miRNAs can be used as ideal potential biomarkers because exosomes protect miRNAs from RNAse activity and increase their stability^9, 46, 47^. An important limitation of our study is that we used synovial fluid samples derived from patients with advanced OA, typically Grade 3 or 4. The differentially expressed exosomal miRNAs seen in our study may not be the same as those required for diagnosis of early stage OA. Similar studies are therefore essential to identify early changes during different stages of OA progression.

In order to determine the biological function of the differentially expressed synovial fluid derived EV miRNAs, KEGG pathway annotation and GO analysis were performed to analyze their target gene pools. KEGG annotation showed that down-regulated miRNAs regulate glycan degradation, cell adhesion molecules (CAMs), and mucin type O-Glycan biosynthesis and up-regulated miRNAs regulate biotin metabolism and thyroid hormone synthesis in both male and female. We also identified a number of differentially regulated KEGG pathways in both male and female but the most prominent were ovarian steroidogenesis and the estrogen signaling pathway. These pathways were affected by up-regulation of miRNAs in female OA. Further detailed analysis showed that 6 miRNAs (miR-181d-3p, miR-3940-3p, miR-155-3p, miR-4532, miR-185-5p, miR-7107-5p) target 9 genes of the ovarian steroidogenesis pathway and the same number of miRNAs (miR-4532, miR-181d-3p, miR-185-5p miR-6865-3p, miR-4459, miR-7107-5p) target 14 genes of the estrogen signaling pathway. We further confirmed that some of these miRNAs (181d-3p, miR-185-5p, miR-7107-5p) target female estrogen signaling pathway genes. These miRNAs regulate expression of ER-α (estrogen receptor- α), ER-β estrogen receptor- β), CYP19 (aromatase cytochrome P450) and CREB-binding protein in human articular chondrocytes.

It has been previously reported that estrogen signaling pathways play important roles in the pathogenesis of female OA^48–50^. After an extensive literature search, we also noted that dysregulated miRNAs in the female OA group are responsive to estrogen signaling in other disease and *in-vitro* models^51, 52^. Some of the EV miRNAs (miR-181d-3p, miR-155-3p, miR-185-5p, miR-3940-3p, miR-4532, miR-7107-5p miR-504-3p, miR-320d, miR-19b-3p and miR-22-3p) up-regulated in female OA synovial fluid were down-regulated in response to estrogen treatment in human and mouse cells^51–54^. The miRNAs (miR-24-3p, miR-26a-5p, miR-200a-3p) down-regulated in female OA samples are known to be elevated with estrogen treatment^51, 52, 55–57^. Based on our findings, we hypothesized that the hormone estrogen might play an important role in EV derived miRNA in the knee joint. In females after menopause, estrogen levels decline^58, 59^ which might affect the EV’s secretion and miRNA cargo. To test this hypothesis, we treated synovial-fibroblast like cells with aromatase inhibitor and analyzed miRNA content of EVs secreted from cells. Our results show that aromatase inhibitor treatment decrease content of miR-26a-5p, miR-146a-5p, miR-328-5p, miR-4654, and increased miR-7107-5p in EVs cargo. Similar trends were noted in female OA miRNA profiling. These differentially regulated miRNAs can be used as gender specific potential biomarkers and/or can also be used for targeting therapeutic interventions. It has been previously reported that estrogen therapy in humans and higher estrogen levels in females have a protective role in OA^60–62^.

GO annotation analysis was performed to identify the regulatory function of the differentially expressed miRNAs. GO pathway annotation analysis showed some common and some gender specific signaling involved in OA. Interestingly, GO pathway annotation indicated that miRNAs targeting TLR and immune signaling pathway were down-regulated in females with OA whereas in males TLR and immune signaling targeting miRNAs were up-regulated. We noted that miRNAs (miR-328-5p, miRNA-26a, hsa-miR-4654, miR-4707-5p, miR-4487, miR-24-3p, miR-6824-5p, miR-4740-5p, miR-8074 and, miR-146a-5p) down-regulated in females with OA have a number of TLR related target genes as per miRNA target prediction software’s (targetscan, miRwalk2.0 and microRNA.org). For example, miRNA-26a targets TLR3 and miR-146a-5p targets three TLR signaling genes (two sites on TLR9, TLR106, TRAF6 and one each on TLR2, and IRAK1). These two miRNAs are significantly down-regulated in female EV miRNAs. Published literature also confirms some of these target genes at mRNA and functional levels (using luciferase assay)^39, 63–65^.

We speculate that in males, OA incidence and severity are significantly lower due to elevated expression of these miRNAs, but in females miRNAs targeting immune and TLR related genes are decreased, possibly reducing their ability to prevent inflammation and the TLR responsive cascade. TLR signaling and inflammation is known to be elevated in OA conditions^66–72^ and that inhibiting or reducing this signaling prevents or reduces OA-like conditions^73^. These signaling pathways are crucial for maintaining normal functioning of female health and their reduction may be one of the reasons for increased incidence of OA in females.

We also demonstrated that the synovial fluid derived EVs efficiently communicate with articular chondrocytes. Our experiments have shown that human articular chondrocytes readily endocytose EVs. Exosomes are endocytosed and release their content into the recipient cells and thereby influence the cellular signaling^8, 10, 11^. We hypothesized that OA derived EVs might affect normal metabolism of articular chondrocytes. This is indeed the case, female OA derived exosomes increase the catabolic activity and decrease anabolic genes when treated to healthy articular chondrocytes. Kato and his co-workers^74^ previously reported that EVs derived from IL-1β stimulated human synovial fibroblasts elevated MMP-13 and ADAMTS-5 expression and decrease COL2A1 and ACAN in articular chondrocytes compared with non-stimulated derived exosomes. Our data strongly suggest the potential important role of these EVs and their miRNAs in female OA pathogenesis. Our study has some limitations; first and most important, we have a small sample size. Similar studies are needed on a larger sample size and at various stages of OA progression. Further studies need to be directed toward confirming miRNA targets in chondrocytes to know the role of differentially expressed miRNAs.

To conclude, this is the first study to demonstrate gender specific miRNA profiling in EVs of synovial fluid in human OA. Synovial fluid derived exosomes play an important role in the pathophysiology of OA. Furthermore, these differentially expressed female miRNAs might be estrogen responsive and play a role in TLR signaling during pathogenesis of OA.

## Electronic supplementary material

Supplementary Information
