# Supplementary material for: Gender-specific differential expression of exosomal miRNA in synovial fluid of patients with osteoarthritis
Source: Sci Rep. 2017 May 17;7:2029. doi: 10.1038/s41598-017-01905-y (PMC5435729; doi:10.1038/s41598-017-01905-y)

Title: Gender-specific differential expression of exosomal miRNA in synovial fluid of patients with osteoarthritis.

Ravindra Kolhe, Monte Hunter, Siyang Liu, Ravirajsinh N.Jadeja, Chetan Pundkar, Ashis K. Mondal, Bharati Mendhe, Michelle Drewry, Mumtaz V. Rojiani, Yutao Liu, Carlos M. Isales, Robert E. Guldberg, Mark W. Hamrick, Sadanand Fulzele

Supplementary figures. **S1**: The unsupervised heat map clustering showing S1 (a) female and S1 (b) male difference between OA and NON-OA samples.

Supplementary Figure. S1(a)

Groups	Patient number
Female NON-OA	23, 29, 70
Female OA	09, 44, 48, 82

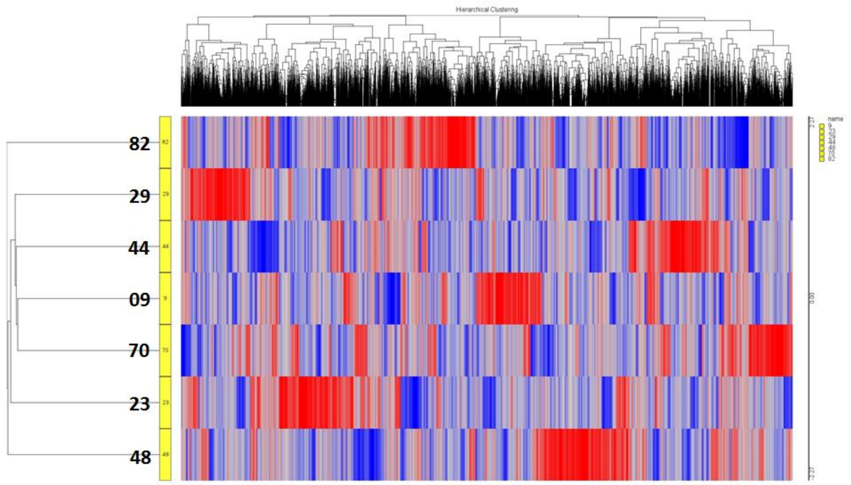

Supplementary Figure. S1(b)

Groups	Patient number
Male NON-OA	65, 69, 181
Male OA	51, 61, 76, 143

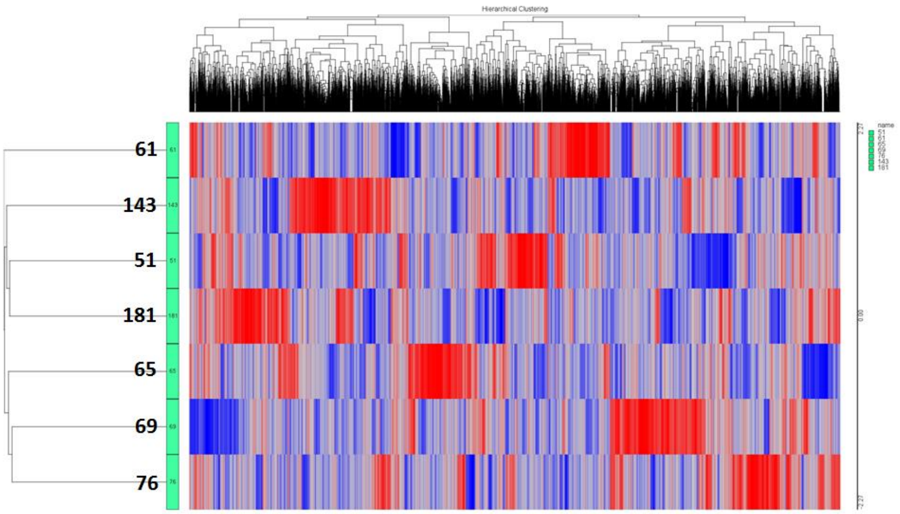

Table S1: Details of the human synovial fluid donor samples used for miRNA-real time PCR.

Gender	Sample type	Number of Samples	Age
**Male**	NON-OA	9	46.40 ± 3.10
	OA	15	50.25 ± 4.80
**Female**	NON-OA	9	44.88 ± 6.99
	OA	18	48.16 ± 4.27

Table S2: Nucleotide sequences of human primers used for RT-PCR.

Gene	Primer Sequence	Reference/Accession number
GADPH	GAC ATC AAG AAG GTG GTG AAG CAG	AF261085
	GCT GTT GAA GTC AGA GGA GAC CAC	
IL-6	CCA ATC TGG ATT CAA TGA GGA G	BT019749
	GCT CTG GCT TGT TCC TCA CTA C	
TNFα	CCC AGG CAG TCA GAT CAT CTT	NM_000594
	TCT CAG CTC CAC GCC ATT	
Human Aggrecan	AGG GCG AGT GGA ATG ATG TT	[Khan et al 2008]
	GGT GGC TGT GCC CTT TTT AC	
Human COL-2A	CTG CAA AAT AAA ATC TCG GTG TTC T	[Khan et al 2008]
	GGG CAT TTG ACT CAC ACC AGT	
CYP19A1	GAT TTT AAC CAC GAT AGC ACT TTC G	[Crider et al 2014]
	CCC TTC TGC GTC GTG TCA T	
n-CoR	TTG GAC TCT TGG ATG TGC C	[Crider et al 2014]
	GCT GAT GAG GAT GTG GAT GG	
CBP	TGT TGA ACA TGA GCC AGA CG	[Crider et al 2014]
	TCA GTC AAC ATC TCC TTC GC	
TIF2	TCT GTG TAT GTG CCA TTC GG	[Crider et al 2014]
	TAA TGC ACA GAT GCT GGC C	
ER-β	CGT AAC ACT TCC GAA GTC GG	[Crider et al 2014]
	TCA CAT CTG TAT GCG GAA CC	
ER-α	GCC AGG CAC ATT CTA GAA GG	[Crider et al 2014]
	AGA CAT GAG AGC TGC CAA CC	

References

1. Khan, W. S., Tew, S. R., Adesida, A. B. & Hardingham, T. E. Human infrapatellar fat pad-derived stem cells express the pericyte marker 3G5 and show enhanced chondrogenesis after expansion in fibroblast growth factor-2. Arthritis Res Ther. 10(4), R74,doi:10.1186/ar2448 (2008).
2. Crider, A., Thakkar, R., Ahmed, A.O. & Pillai, A. Dysregulation of estrogen receptor beta (ERβ), aromatase (CYP19A1), and ER co-activators in the middle frontal gyrus of autism spectrum disorder subjects. Mol Autism. 9, 5(1), 46 (2014).

Table S3. Selected KEGG biological pathways potentially affected by (a) miRNAs down-regulated in male OA, (b) miRNAs up-regulated in male OA, (c) miRNAs down-regulated in female OA , and (d) miRNAs up-regulated in female OA.

Table S4. Selected GO biological pathways potentially affected by (a) miRNAs down-regulated in male OA, (b) miRNAs up-regulated in male OA, (c) miRNAs down-regulated in female OA , and (d) miRNAs up-regulated in female OA.

Table. S3: (a) Selected KEGG pathways affected by miRNAs down-regulated in male OA.

**KEGG pathway**	**p-value**	**Number of targets genes**	**miRNAs involved**
**Common Pathway**			
Other glycan degradation	0.0004	3	3
Cell adhesion molecules (CAMs)	0.0007	29	8
Mucin type O-Glycan biosynthesis	0.0238	7	5
**Differentially regulated Pathway**			
Glycosphingolipid biosynthesis - globo series	0.0208	6	3
Glycosphingolipid biosynthesis - lacto and neolacto series	3.69E-19	9	5
Glycerophospholipid metabolism	1.45E-07	20	7
Ether lipid metabolism	0.0004	14	5
alpha-Linolenic acid metabolism	0.0010	11	5
Metabolism of xenobiotics by cytochrome P450	0.0409	11	7
Linoleic acid metabolism	0.0460	11	5

Table. S3: (b) Selected KEGG pathways affected by miRNAs up-regulated in male OA

**KEGG pathway**	**p-value**	**Number of targets genes**	**miRNAs involved**
**Common Pathway**			
Biotin metabolism	0.00013	1	1
Thyroid hormone synthesis	0.01694	10	5
**Differentially regulated Pathway**			
Fatty acid elongation	7.02E-07	3	3
Fatty acid degradation	0.000473	6	4
Cholinergic synapse	0.002554	25	7
GABAergic synapse	0.003157	19	6
Glutamatergic synapse	0.004433	25	7
Retrograde endocannabinoid signaling	0.007197	19	6
Ras signaling pathway	0.007197	44	8

Table. S3: (c) Selected KEGG pathways affected by miRNAs down-regulated in female OA.

**KEGG pathway**	**p-value**	**Number of targets genes**	**miRNAs involved**
**Common Pathway**			
Other glycan degradation	0.00041	9	3
Cell adhesion molecules (CAMs)	0.02091	14	6
Mucin type O-Glycan biosynthesis	8.30E-05	5	4
**Differentially regulated Pathway**			
Fatty acid metabolism	6.16E-14	2	2
Axon guidance	0.00625	4	3
Vasopressin-regulated water reabsorption	0.00765	30	8
ECM-receptor interaction	0.00956	12	4
Long-term depression	0.00956	13	8
Fatty acid elongation	0.02091	24	10
Glycosphingolipid biosynthesis - ganglio series	0.03982	2	2
ErbB signaling pathway	0.03982	3	3
Galactose metabolism	0.03982	18	5
MAPK signaling pathway	0.04533	5	4

Table. S3: (d) Selected KEGG pathways affected by miRNAs up-regulated in female OA

**KEGG pathway**	**p-value**	**Number of targets genes**	**miRNAs involved**
**Common Pathway**			
Biotin metabolism	1.79E-05	1	1
Thyroid hormone synthesis	4.73E-06	14	5
**Differentially regulated Pathway**			
Ovarian steroidogenesis	***0.026590***	***9***	***6***
Estrogen signaling pathway	***0.026590***	***14***	***6***
Other types of O-glycan biosynthesis	9.00E-07	8	8
Glycosphingolipid biosynthesis - lacto and neolacto series	4.73E-06	5	3
Primary bile acid biosynthesis	0.003953	3	1
Amphetamine addiction	0.017037	10	6
Tyrosine metabolism	0.017227	6	4
Synaptic vesicle cycle	0.026590	13	7
ECM-receptor interaction	0.027953	9	5

Table. S4: (a) Selected GO pathways affected by miRNAs down-regulated in Male OA.

GO pathway	p-value	Number of targets genes	miRNAs involved
**Common pathway**			
catabolic process	1.26E-09	249	12
cellular protein metabolic process	1.79E-09	71	13
neurotrophin TRK receptor signaling pathway	1.06E-07	41	13
cellular protein modification process	3.28E-06	266	13
glycosaminoglycan metabolic process	7.27E-06	23	8
response to stress	0.000221	263	13
Fc-epsilon receptor signaling pathway	0.000567	23	11
O-glycan processing	0.000572	13	6
cell junction assembly	0.000749	14	7
glycerophospholipid biosynthetic process	0.001226	17	7
epidermal growth factor receptor signaling pathway	0.005497	30	11
extracellular matrix organization	0.008976	52	12
nucleic acid binding transcription factor activity	0.024867	109	12
cytoskeletal protein binding	0.032862	89	11
activation of phospholipase C activity	0.039726	12	6
**Differentially regulated Pathway**			
organelle	3.07E-38	1171	13
cellular nitrogen compound metabolic process	1.16E-23	583	13
small molecule metabolic process	2.79E-17	319	13
biosynthetic process	3.27E-15	487	13
post-translational protein modification	3.34E-09	35	11
phosphatidylserine acyl-chain remodeling	7.38E-05	8	4
phosphatidylethanolamine acyl-chain remodeling	7.38E-05	9	4
lysosomal lumen	0.000305	15	4
sulfur compound metabolic process	0.000567	44	11
nucleobase-containing compound catabolic process	0.001226	107	12
phosphatidylcholine acyl-chain remodeling	0.001712	9	5
mitotic cell cycle	0.002028	47	12
cell death	0.0124	107	12
chondroitin sulfate metabolic process	0.012575	10	4
protein N-linked glycosylation via asparagine	0.024867	16	9
phospholipid metabolic process	0.02867	25	9
activation of signaling protein activity involved in unfolded protein response	0.032401	11	7
macromolecular complex assembly	0.033271	97	13
keratan sulfate metabolic process	0.036016	7	3
leukocyte migration	0.038383	18	9
glycosaminoglycan catabolic process	0.039726	7	5
cellular lipid metabolic process	0.039726	19	9

Table. S4: (b) Selected GO pathways affected by miRNAs up-regulated in Male OA

GO pathway	p-value	Number of targets genes	miRNAs involved
**Common Pathway**			
organelle	4.26E-30	1056	10
ion binding	4.43E-24	686	10
cellular nitrogen compound metabolic process	1.54E-17	518	10
neurotrophin TRK receptor signaling pathway	4.16E-12	47	9
biosynthetic process	4.10E-08	416	10
catabolic process	3.09E-05	211	10
cellular protein modification process	0.000102	239	10
symbiosis, encompassing mutualism through parasitism	0.000399	59	10
nucleic acid binding transcription factor activity	0.000489	112	10
nucleobase-containing compound catabolic process	0.006273	96	8
**Differentially regulated Pathway**			
Fc-epsilon receptor signaling pathway	1.47E-06	27	9
cellular component assembly	6.02E-05	149	10
immune system process	0.003928	173	10
toll-like receptor TLR1:TLR2 signaling pathway	0.03092	10	7
toll-like receptor TLR6:TLR2 signaling pathway	0.03092	10	7
response to stress	0.001292	238	10
cytoskeletal protein binding	0.000102	97	10
cell-cell signaling	0.000138	85	9
post-translational protein modification	0.000248	25	8
energy reserve metabolic process	0.000457	19	6
apoptotic signaling pathway	0.000561	25	7
epidermal growth factor receptor signaling pathway	0.001556	30	9
transmembrane transporter activity	0.002909	123	8
macromolecular complex assembly	0.002996	97	9
cellular protein metabolic process	0.003322	49	9
vitamin metabolic process	0.006702	13	6
fibroblast growth factor receptor signaling pathway	0.01125	27	9
cellular response to glucagon stimulus	0.014311	9	5
cell death	0.015627	99	10
extracellular matrix disassembly	0.023831	16	7
phosphatidylinositol-mediated signaling	0.028848	20	9
generation of precursor metabolites and energy	0.03092	40	8
cellular component disassembly involved in execution phase of apoptosis	0.038484	8	5
toll-like receptor 4 signaling pathway	0.041607	14	7
MyD88-independent toll-like receptor signaling pathway	0.042462	11	6
vesicle-mediated transport	0.049537	115	9

Table. S4: (c) Selected GO pathways affected by miRNAs down-regulated in female OA.

**GO pathway**	**p-value**	**Number of targets genes**	**miRNAs involved**
**Common Pathway**			
neurotrophin TRK receptor signaling pathway	5.97E-17	57	12
nucleic acid binding transcription factor activity	4.16E-10	148	13
Fc-epsilon receptor signaling pathway	7.47E-06	27	11
cellular protein modification process	1.47E-05	266	14
catabolic process	5.44E-05	227	14
epidermal growth factor receptor signaling pathway	9.35E-05	35	11
cellular protein metabolic process	0.000196	57	12
cell junction assembly	0.000653	14	7
cytoskeletal protein binding	0.00156	98	12
O-glycan processing	0.002094	12	7
response to stress	0.002697	257	14
activation of phospholipase C activity	0.013489	13	5
glycosphingolipid metabolic process	0.024297	11	7
extracellular matrix organization	0.028039	50	11
glycerophospholipid biosynthetic process	0.029186	14	7
**Differentially regulated Pathway**			
cell-cell signaling	3.84E-09	108	13
extracellular matrix disassembly	3.37E-05	23	8
toll-like receptor 10 signaling pathway	***0.002094***	***12***	***5***
TRIF-dependent toll-like receptor signaling pathway	***0.002477***	***13***	***5***
toll-like receptor TLR1:TLR2 signaling pathway	***0.003676***	***12***	***5***
toll-like receptor 9 signaling pathway	***0.004682***	***13***	***5***
protein binding transcription factor activity	0.000168	66	11
fibroblast growth factor receptor signaling pathway	0.000832	32	11
energy reserve metabolic process	0.000944	19	6
inositol phosphate metabolic process	0.000168	12	7
immune system process	***0.006128***	***187***	***14***
regulation of insulin secretion	0.002477	22	6
toll-like receptor 5 signaling pathway	***0.006679***	***12***	***5***
MyD88-independent toll-like receptor signaling pathway	***0.007024***	***13***	***5***
regulation of defense response to virus by virus	0.013557	7	6
macromolecular complex assembly	0.018813	100	14
toll-like receptor 3 signaling pathway	***0.025012***	***13***	***5***
intrinsic apoptotic signaling pathway	0.034503	13	5
stress-activated MAPK cascade	0.038929	10	5

Table. S4: (d) Selected GO pathways affected by miRNAs up-regulated in female OA

GO pathway	p-value	Number of targets genes	miRNAs involved
**Common Pathway**			
organelle	3.14E-29	813	13
cellular nitrogen compound metabolic process	1.58E-17	406	13
ion binding	3.54E-17	507	13
biosynthetic process	2.53E-12	343	12
nucleic acid binding transcription factor activity	5.75E-06	97	12
symbiosis, encompassing mutualism through parasitism	6.70E-06	53	11
catabolic process	3.61E-05	164	12
nucleobase-containing compound catabolic process	6.77E-05	85	12
neurotrophin TRK receptor signaling pathway	0.001505	25	10
cellular protein modification process	0.004634	175	12
**Differentially regulated Pathway**			
small molecule metabolic process	2.35E-13	225	13
gene expression	5.52E-07	58	12
cellular lipid metabolic process	0.000291	20	10
cell death	0.001481	84	12
mitotic cell cycle	0.007295	35	10
protein complex	0.009041	294	12
cytoskeletal protein binding	0.009879	69	12
cellular protein metabolic process	0.014747	38	12
enzyme binding	0.017307	104	13
clathrin-sculpted monoamine transport vesicle membrane	0.029929	3	2
molecular_function	0.033726	1319	13

Figure 1(b) Western Blot

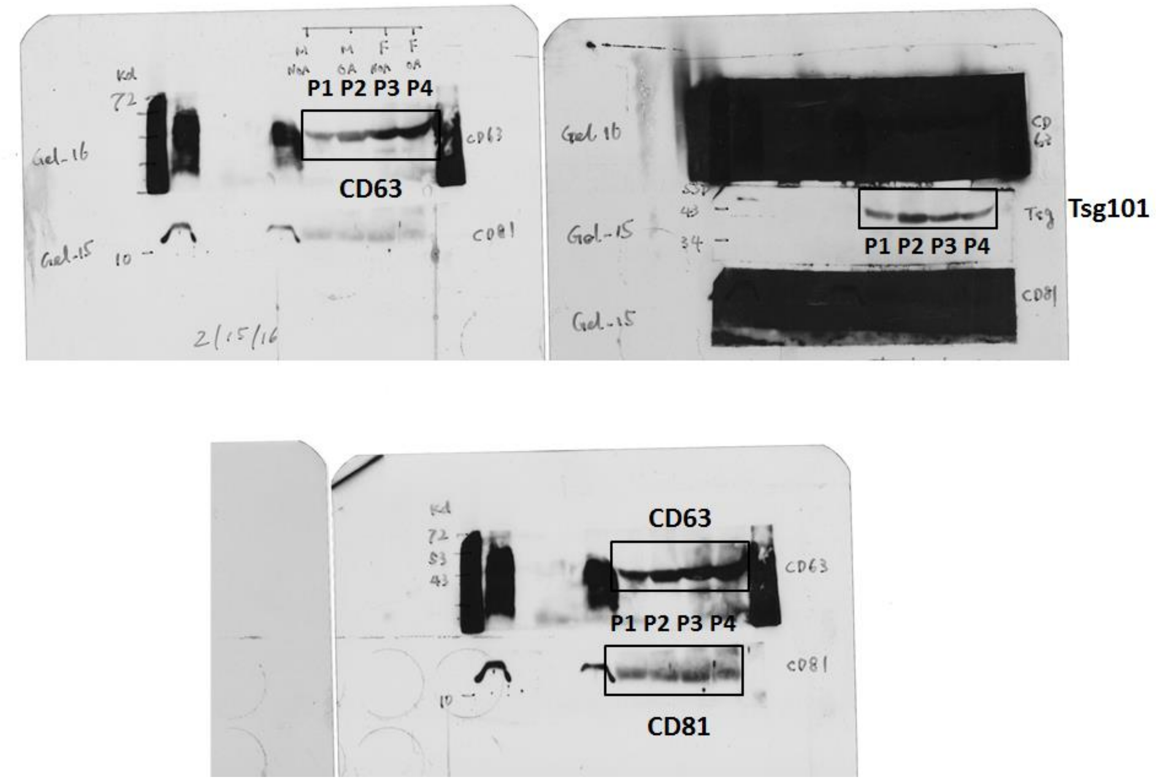

Figure 8(b) Gelatin zymography

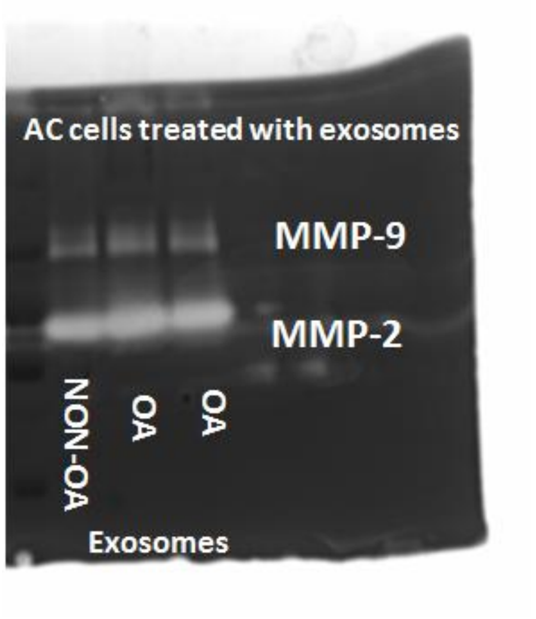

Supplement: Supplementary file 1 — Supplementary Information [file 41598_2017_1905_MOESM1_ESM.pdf]
